# Racial disparities in the diagnosis of disruptive behavior disorders: a U.S. national inpatient sample analysis

**DOI:** 10.3389/fpsyt.2024.1425559

**Published:** 2024-09-12

**Authors:** William M. Savage, Sidney A. Saint-Hilaire, Mansi Shah, Claudia Lugo-Candelas

**Affiliations:** ^1^ Vagelos College of Physicians and Surgeons, Columbia University, New York, NY, United States; ^2^ Department of Psychiatry, Columbia University, New York, NY, United States; ^3^ Department of Child and Adolescent Psychiatry, New York State Psychiatric Institute, New York, NY, United States

**Keywords:** child and adolescent mental health, diagnostic disparities, disruptive behavior disorders, attention deficit hyperactivity disorder, health disparities

## Abstract

Disruptive behavior disorders (DBDs) are comprised of conduct disorder and oppositional defiant disorder. Limited literature exists on the demographics of patients diagnosed with these conditions. Despite the clinical overlap of DBDs and attention-deficit hyperactivity disorder (ADHD), there is a stark divergence in the treatment and societal accommodation for these two diagnoses, amplifying the importance of this diagnostic challenge. Thus, potential diagnostic differences must be urgently and rigorously explored. Small, regional studies have reported potential “racial” disparities in the diagnosis of DBDs, especially when compared to the diagnosis of ADHD. Our study uses the National Inpatient Sample (NIS) database to provide the largest, most comprehensive investigation of “racial” disparities in the diagnosis of DBDs. Discharge data from 700,770 pediatric inpatients (mean age = 9.32 years) were included in this analysis. To explore potential disparities among children with symptoms in this area of clinical overlap, we assessed the relationship of “race” and the diagnosis of DBDs. Among the subgroup of pediatric inpatients diagnosed with ADHD or a DBD, Native American (OR = 2.18; 95% = 1.76, 2.70), Asian (OR = 1.88, 95% = 1.56, 2.26), Black (OR = 1.40; 95% = 1.32, 1.48), and Hispanic (OR = 1.20; 95% = 1.12, 1.28) “race” correlated with DBD diagnosis. By highlighting these diagnostic disparities, this study raises essential questions about race and the diagnosis of DBDs.

## Introduction

Healthcare disparities remain among the most traumatic of injustices in the United States. Despite growing awareness and innumerable efforts to address such inequities, healthcare disparities persist (1–4). The need to address disparities in our healthcare system has become even more acute with a rapidly changing and increasingly diverse population, wherein over half of children under 16 years old in the United States now identify as a “racial” or ethnic minority (5). Further, the emergence of machine learning and its steady incorporation into the clinical sphere is of great concern, as these artificial intelligence algorithms learn from historical, oftentimes severely biased, data, and thus, may perpetuate the disparities that we now seek to alleviate (6).

The advent of open medical records now grants patients access to previously undisclosed clinical notes and diagnoses. Within psychiatry, this advancement further raises the importance of clinician language choice and diagnostic precision. Now, the impact of inappropriate diagnoses, even between psychiatric disorders with substantial overlap, is significantly magnified. Such clinical misjudgments now have greater potential to undermine the clinician-patient relationship and erode patient trust in the medical system, highlighting the need to explore the dynamics underlying diagnostic disparities.

While “racial” disparities in psychiatric diagnoses are well-documented (7–10), limited literature exists on the demographics of patients diagnosed with disruptive behavior disorders (DBDs) (11–15). These DBDs, comprised of oppositional defiant disorder and conduct disorder, are rife with educational and psychosocial consequences. There is significant clinical overlap between these disorders and attention-deficit hyperactivity disorder (ADHD), and the tendency of these disorders to co-occur has been reported (16). A diagnosis of a DBD rather than ADHD can severely reduce access to psychiatric and educational resources (12). Thus, the diagnostic dilemma involving DBDs and ADHD can serve as a pivotal moment in a child’s trajectory.

Importantly, “race” is a social construct without biologic basis, and thus has limited utility in a scientific analysis. Yet, “racial” disparities in the diagnosis of DBDs and ADHD, if present, perpetuate cycles of systemic racism that often underlie healthcare inequity (17). While “racial” disparities in the diagnosis of ADHD have been well-documented (10, 18, 19), a more thorough investigation of potential disparities in the diagnosis of DBDs is warranted.

The evidence that “racial” disparities exist in the diagnosis of DBDs (20, 21) would be markedly strengthened by a robust, nationwide analysis. We report herein such a study using data from the National Inpatient Sample (NIS) database. Further, while prior studies in this area have focused on outpatient psychiatry, this study utilizes inpatient data, thereby highlighting potential disparities in an especially vulnerable population. The following study thoroughly explores demographic characteristics of patients diagnosed with DBDs compared with those diagnosed with ADHD, providing needed insight into the diagnostic disparities that may perpetuate undue stigma and trauma on populations for whom just, equitable healthcare remains elusive.

## Materials and methods

Hospitalized pediatric patients (<18 years old) registered in the NIS database (2017-2019) were included. The NIS database is the largest inpatient database in the United States, providing detailed socioeconomic (age, sex, race, insurance type, zip-code median income) and encounter-level information, including International Classification of Diseases (ICD-10) diagnostic and procedural codes. The diagnoses of interest were defined according to ICD-10-CM codes, as follows: attention-deficit hyperactivity disorders (F900, F901, F902, F908, F909); and disruptive behavior disorders, which are comprised of conduct disorders (F910, F911, F912, F918, F919) and oppositional defiant disorder (F913). The NIS database inadequately divides “race” into six groups (e.g., including Hispanic ethnicity as a “race”). These groups are Asian, Black, Hispanic, Native American, Other, and White. Multiple logistic regression analysis, odds ratios, and figures were generated using R (R Core Team, 2023). Two sets of regressions were performed. The association of “race” with DBD and ADHD diagnoses was explored (1) among all children and (2) among the subset of children who were diagnosed with either a DBD or ADHD.

## Results

**Figure 1:**
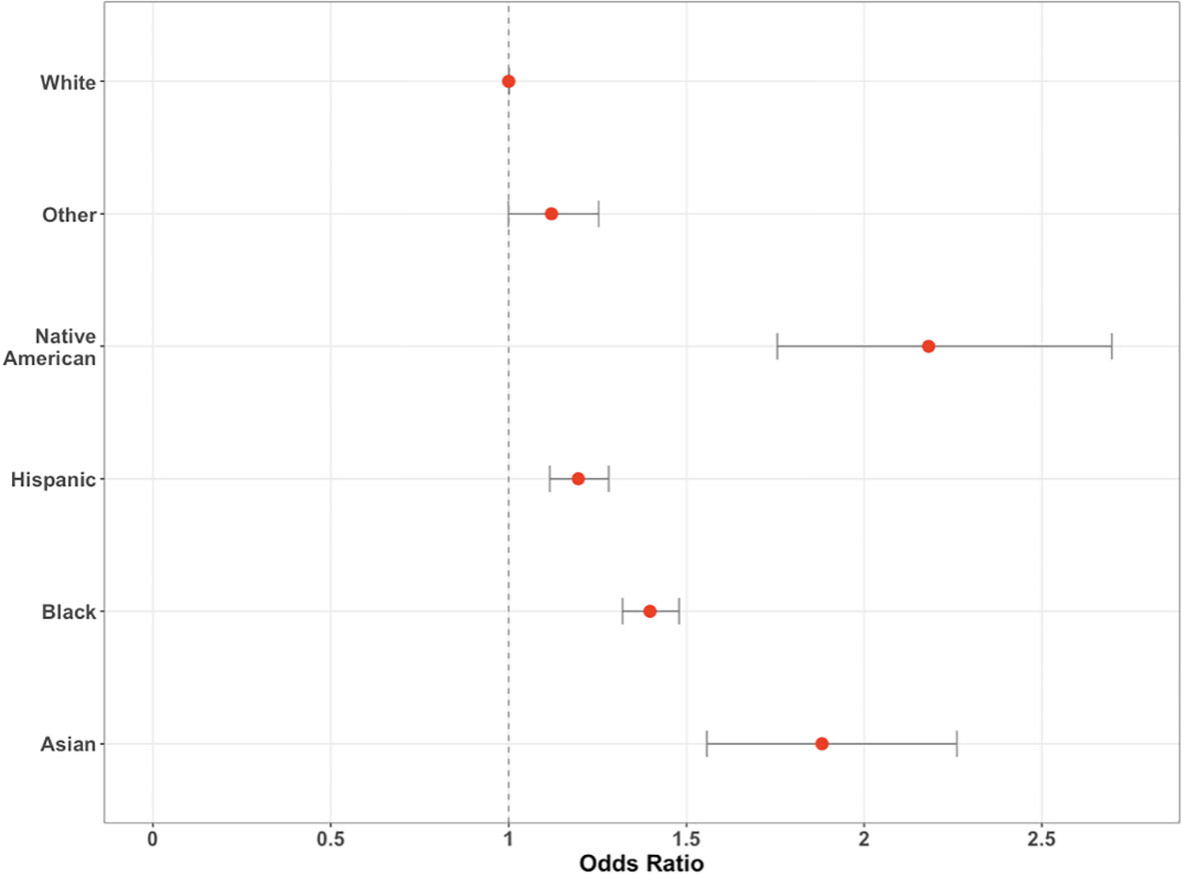
Among pediatric inpatients diagnosed with either a DBD or ADHD, after controlling for age, gender, zip code median income, and insurance type, odds of a pediatric inpatient of a given “race” receiving a diagnosis of a DBD, relative to a pediatric inpatient of White “race.” Error bars represent 95% confidence intervals.

Discharge data from 700,770 pediatric inpatients (mean age = 9.32 years) were included in this analysis. Within this dataset, 22,587 inpatients (mean age = 12.70 years) were diagnosed with a DBD, and 57,980 inpatients (mean age = 12.50 years) were diagnosed with ADHD. All analyses controlled for age, gender, insurance type, and zip-code-median income. Relative to White “race”, Black “race” correlated with DBD diagnosis (OR = 1.05; 95% = 1.02, 1.09) and inversely correlated with ADHD diagnosis (OR = 0.80; 95% = 0.79, 0.82). To explore potential disparities among children with symptoms in this area of clinical overlap, among the subgroup of pediatric inpatients diagnosed with ADHD or a DBD, Native American (OR = 2.18; 95% = 1.76, 2.70), Asian (OR = 1.88, 95% = 1.56, 2.26), Black (OR = 1.40; 95% = 1.32, 1.48), and Hispanic (OR = 1.20; 95% = 1.12, 1.28) “race” correlated with DBD diagnosis (**Figure 1**). Additional analyses including the subgroup of patients with both a DBD and ADHD are shown in **Supplementary Figure 1**.

## Discussion

To our knowledge, our study is the largest and most comprehensive investigation regarding “racial” disparities in the diagnosis of DBDs and ADHD in inpatient pediatrics. DBDs and ADHD are intended to be diagnosed in patients < 18 years of age. Thus, the study sample included only patients under 18 years old. Patients who were diagnosed as having both a DBD and ADHD were excluded from the primary analysis.

This study, involving 700,770 pediatric inpatients hospitalized in the years 2017-2019, supports prior literature suggesting that White “race” may serve as a “protective factor” against the diagnosis of a DBD, while belonging to a “racial” minority may serve as a “risk factor” for the same diagnoses. It should first be noted that any “race-based” analysis has inherent limitations. “Race” and ethnicity are social constructs without scientific meaning or genetic basis (1); in fact, the human genome project has revealed that there is more genetic heterogeneity within “racial” groups than between “racial” groups (22). Assignment of “race” does not encompass the complexity and diversity of a patient’s lived experience, yet efforts to explore health disparities necessarily rely on metrics such as “race” in order to identify areas of inequity (17). The National Inpatient Sample, for instance, documents the patient’s race in an incomplete and – due to the vastness of the dataset – likely inconsistent manner. This database uses the term “race” when “ethnicity” may be more appropriate, such as when referring to patients of Hispanic descent; however, addressing or correcting this verbiage is beyond the scope of this study. Describing a patient’s “race” as one of six categories, as is done here, is woefully inadequate: If “race” and ethnicity must be used, more specific identifying terms would be preferable (17).

Despite these limitations, the National Inpatient Sample database can provide powerful insight into national trends in inpatient clinical care. Designed to be representative of national hospital inpatient data, the National Inpatient Sample, a component of the United States’ Department of Health and Human Services (HHS) Healthcare Cost and Utilization Project (HCUP), provides demographic, diagnostic, treatment, and outcome data on over 7 million inpatients annually. The expanse of this dataset allows this study to provide the most substantial evidence to date that “racial” disparities exist in the diagnosis of DBDs when compared to the diagnosis of ADHD. Importantly, the size of this study sample allows careful analysis of groups often excluded from underpowered studies due to sample size concerns, such as Native Americans.

The finding that some minority groups are more commonly diagnosed with DBDs supports the findings of smaller, prior studies (20, 21). This study’s direct comparison of DBD and ADHD diagnosis highlights an inflection point in pediatric psychiatry: For a patient with clinical symptoms that reside in the intermediate zone between DBD and ADHD, the clinician’s diagnosis will often precipitate markedly divergent pathways. The patient with an ADHD diagnosis will likely receive medications, psychotherapy, and academic accommodations; thus, the inappropriate diagnosis of ADHD – whether under- or over-diagnosis – has clear clinical and ethical consequences. In contrast, the patient diagnosed with a DBD without a concurrent ADHD diagnosis is less likely to receive these treatments and accommodations. Furthermore, given the associations between DBD and aggressive, antisocial behavior, such a diagnosis comes with the risk of social ostracization and stigmatization that follows children into the classroom, the courtroom and beyond (23, 24). Knowing this, if the clinical decision-making in these moments of relative ambiguity is – as these data strongly suggest – racially inequitable, these diagnoses propagate and likely exacerbate “racial” disparities not just in pediatric healthcare, but in a myriad of downstream arenas, including academic and legal settings.

While the use of the National Inpatient Sample dataset allows for the most exhaustive investigation of DBD to date, the breadth of the dataset hinders its depth; we are not able to assess individual-level clinical data such as self-reported clinical need and more granular assessments of covariates like household income. Our analysis provides critical epidemiological findings which would be complemented by smaller studies with detailed patient-level data and more close phenotyping of symptoms and associated impairment. For instance, because DBDs are multifactorial in origin, with a variety of environmental factors such as disorganized attachment and unstable home environments contributing to their development (25), one must consider whether minority children are more likely to experience psychosocial risk factors for DBD development. While this dataset does not contain the detailed, qualitative data required to control for such psychosocial factors, our analysis controls for socio-economic status, in the form zip-code-median income. In this way, while we must consider the potential role of these psychosocial factors in contributing to the observed diagnostic disparities, our data suggest strongly that socio-economic status and related psychosocial factors do not contribute significantly to the observed diagnostic disparities reported here. Furthermore, this dataset is not equipped to assess the divergent treatment pathways and quality of care associated with ADHD versus DBDs. Importantly, the ongoing National Institutes of Health (NIH) Adolescent Brain Cognitive Development (ABCD) Study is a longitudinal study involving 11,880 children, and thus may provide robust clinical and radiographic data regarding the effects of various treatment regimens on behavioral disturbances over time (26–29).

Our study raises essential questions about unconscious bias and cultural humility. Unconscious bias persists in medicine and will continue to persist (30, 31). Furthermore, implicit and systemic bias are well-established contributors to inequities in healthcare (32, 33). To address disparities in the diagnosis of DBDs, training psychiatrists and other healthcare providers to acknowledge their biases may be a fruitful first step (12). Relatedly, enhanced training in cultural humility may help clinicians better recognize the complex cultural landscapes which underlie clinical encounters and likely contribute to the disparities documented in this study (34). This study illuminates the relationship between “race” and the diagnosis of DBDs. Future work must explore potential avenues to ameliorate these disparities.

## Data Availability

The original contributions presented in the study are included in the article/**Supplementary Material**. Further inquiries can be directed to the corresponding author.

## Supplementary material

Supplementary Figure 1 Among pediatric inpatients diagnosed with a DBD and/or ADHD, after controlling for age, gender, zip code median income, and insurance type, odds of a pediatric inpatient of a given “race” receiving a diagnosis of a DBD, relative to a pediatric inpatient of White “race.” Error bars represent 95% confidence intervals.
