# Supplementary figures and images for: Racial disparities in the diagnosis of disruptive behavior disorders: a U.S. national inpatient sample analysis
Source: Front Psychiatry. 2024 Sep 12;15:1425559. doi: 10.3389/fpsyt.2024.1425559 (PMC11424396; doi:10.3389/fpsyt.2024.1425559)

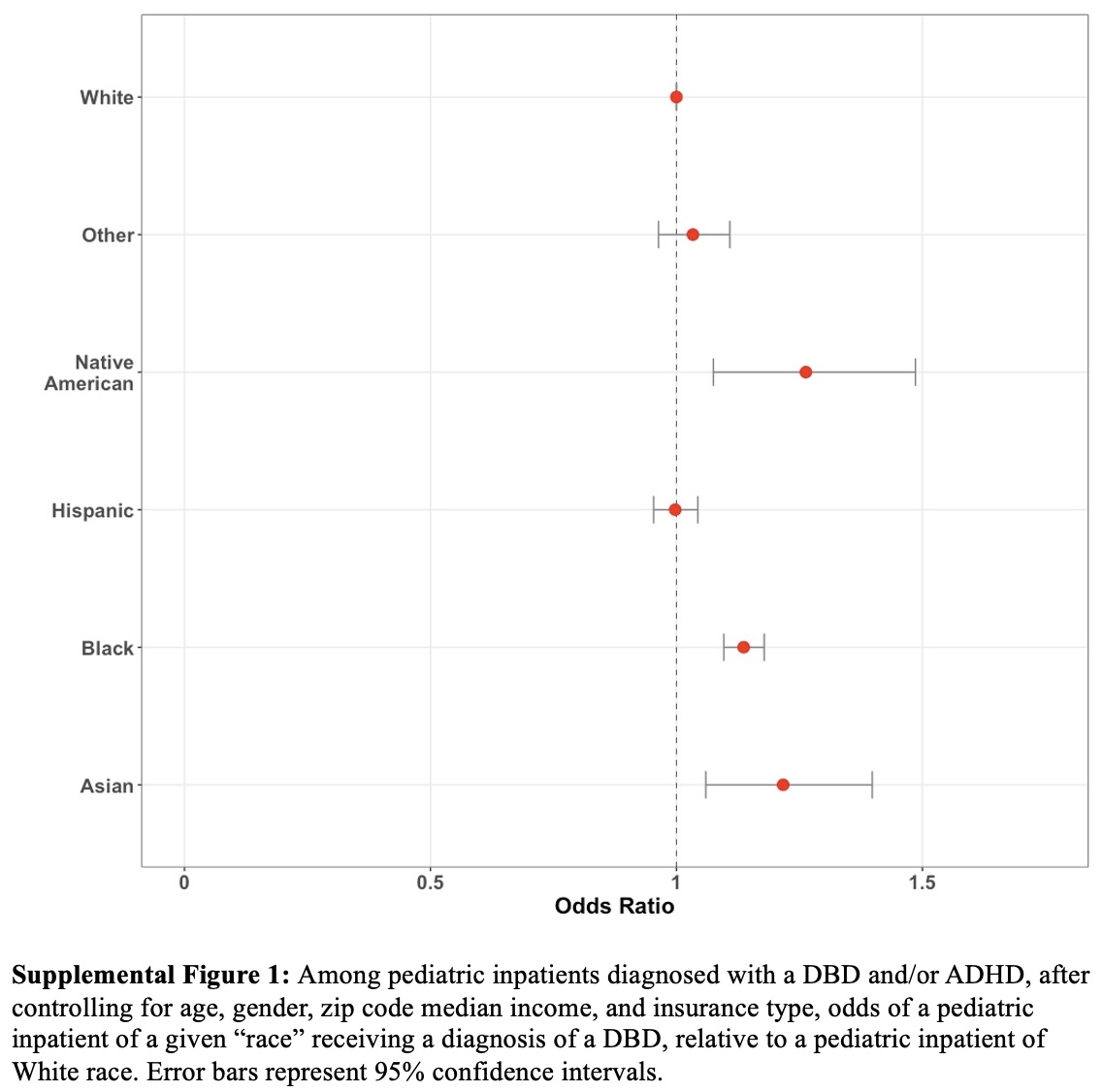

Supplement: Supplementary Figure 1 — Among pediatric inpatients diagnosed with a DBD and/or ADHD, after controlling for age, gender, zip code median income, and insurance type, odds of a pediatric inpatient of a given “race” receiving a diagnosis of a DBD, relative to a pediatric inpatient of White “race.” Error bars represent 95% confidence intervals. [file Image1.jpeg]
